# Supplementary material for: NAD(H) homeostasis underlies host protection mediated by glycolytic myeloid cells in tuberculosis
Source: Nat Commun. 2023 Sep 6;14:5472. doi: 10.1038/s41467-023-40545-x (PMC10482943; doi:10.1038/s41467-023-40545-x)
Supplement: Supplementary file 1 — Supplementary Information [file 41467_2023_40545_MOESM1_ESM.pdf]

Supplementary Information

NAD(H) homeostasis underlies host protection mediated by glycolytic myeloid cells in tuberculosis

Hayden T. Pacl, Krishna C. Chinta, Vineel P. Reddy, Sajid Nadeem, Ritesh R. Sevalkar, Kievershen Nargan, Kapongo Lumamba, Threnesan Naidoo, Joel N. Glasgow, Anupam Agarwal and Adrie J. C. Steyn

Contents:

- **Table S1**
- **Figures S1-S7**

Table S1. Clinical characteristics of human subjects.

#	Patient #	Age	Sex	Macroscopic and microscopic features	Type of resection
1	AS314	68	M	Lung tissue showed large areas of granulomatosis inflammation and granulation tissue. Some granulomas appear healed. Acid-fast bacilli are present, and chronic inflammatory cell infiltrates are noted. Large numbers of intra-alveolar foamy macrophages are present.	Left upper lobe lobectomy
2	AS211	30	F	Specimen demonstrates cavitational and miliary tuberculosis as is evident by areas of fibrocaseous necrosis, interstitial fibrosis, fibro-calcified nodules and presence of acid-fast bacilli. Features of lymphoid interstitial pneumonia are present.	Left pneumonectomy
3	AS134	38	F	Cut sections of the left lung show bronchiectasis and caseative necrosis. Foci of suppuration are noted including a 2 cm cavity in the upper lobe. Microabscess formation is noted, and acid-fast bacilli are present.	Left pneumonectomy

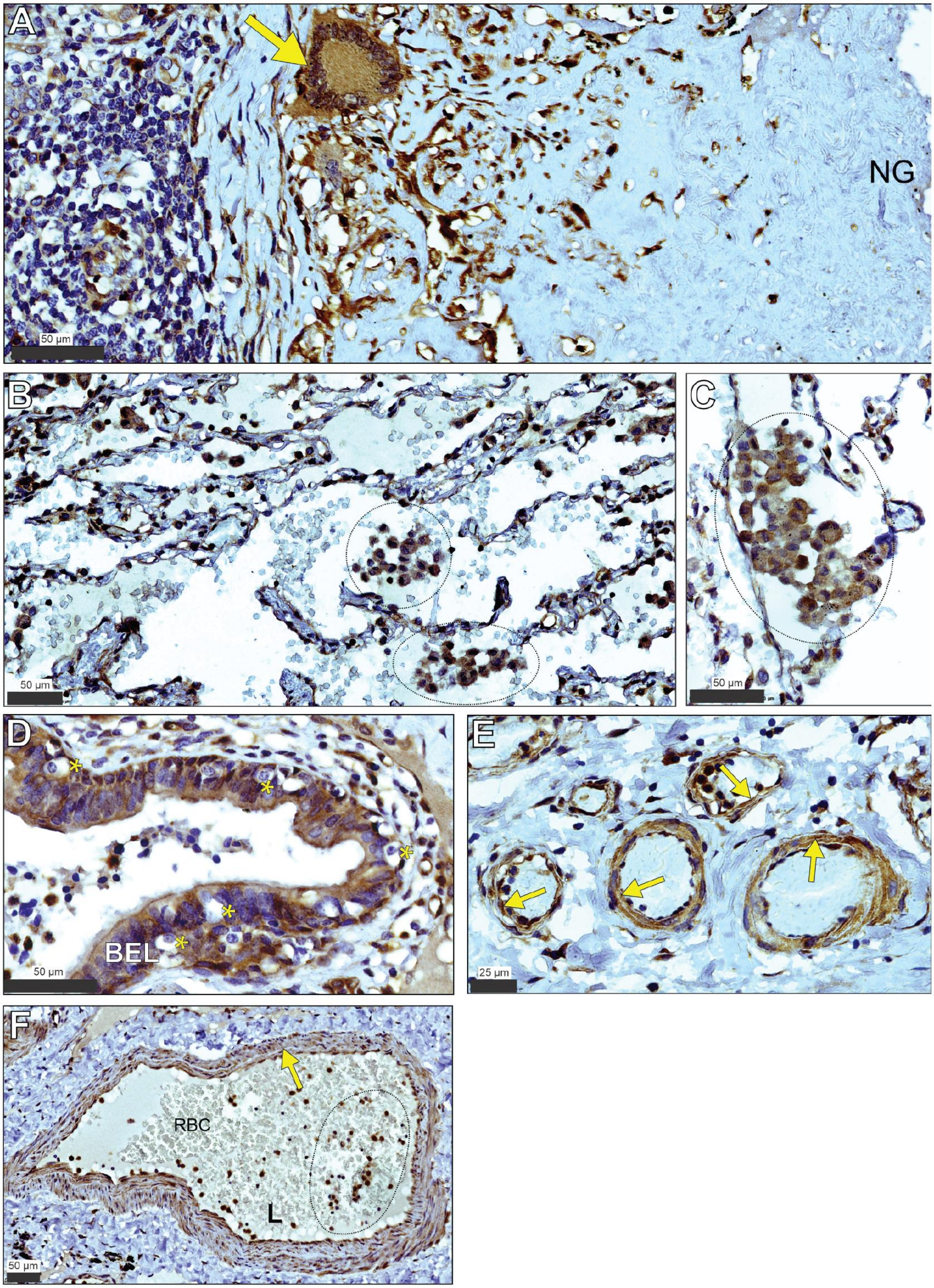

Figure S1. LDHA expression in alveolitis, giant cells, bronchial epithelial cells, and vasculature in the human TB lung. (A) High-power image of a giant cell stained for LDHA (yellow arrow) in the context of a necrotizing granuloma (NG). Low- **(B)** and **(C)** medium-power magnification images of LDHA immunostaining in

leukocytes in the alveoli (circled regions) of a TB patient. **(D)** shows the bronchial endothelial layer (BEL) stained for LDHA, as well as unstained cells (yellow asterisks) that represent mucus-producing cells. **(E, F)** Small **(E)** and large **(F)** vessel walls stained for LDHA expression (yellow arrows). **(F)** also shows robust LDHA staining in leukocytes within the lumen of the depicted vessel (circled region). Tissue staining and staining controls obtained from three TB patients (Table S1) were performed at least three times independently post optimization.

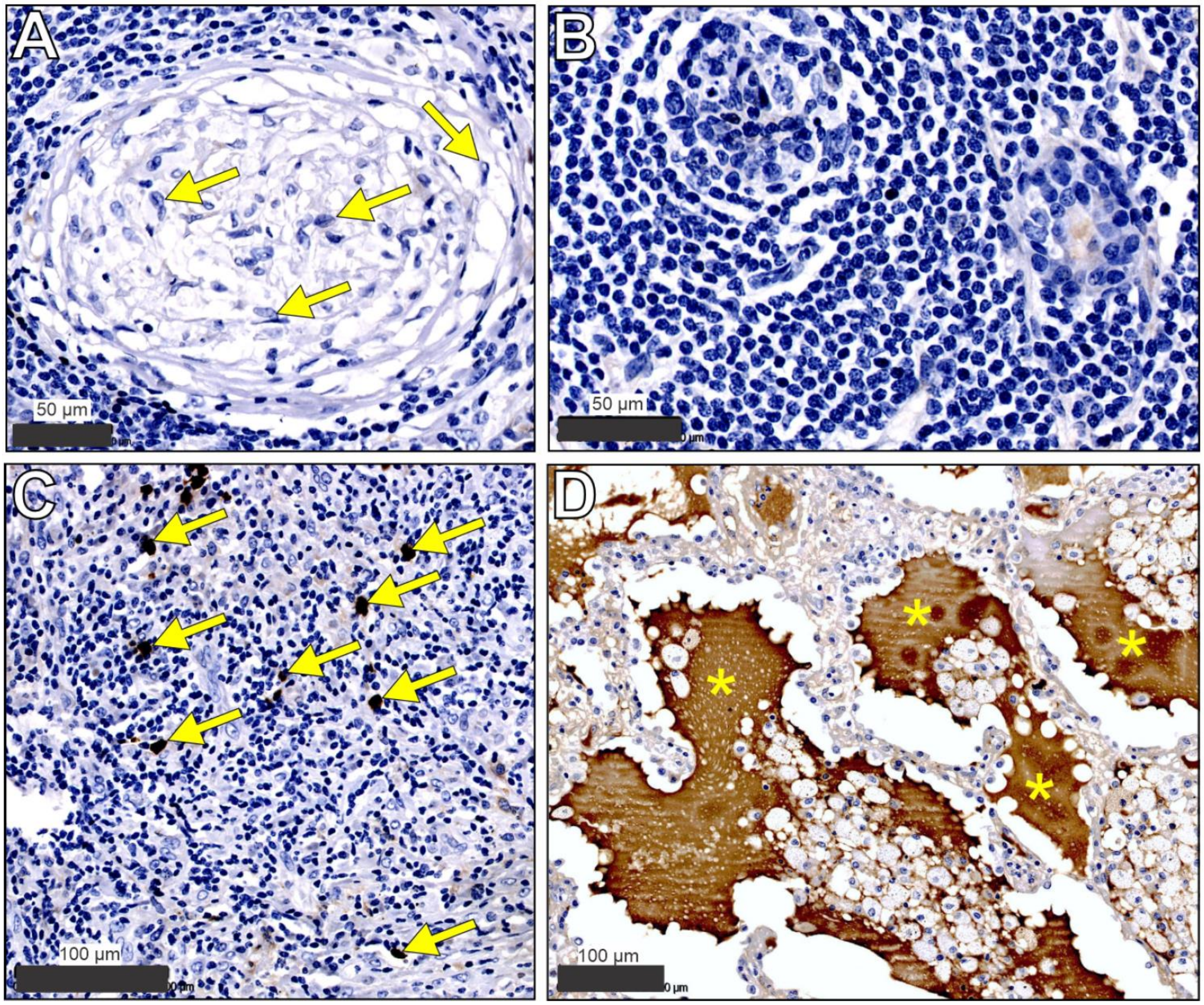

Figure S2. Use of IgG4 as an isotype control for IHC. High power depiction of a granuloma (**A**) and lymphocytic aggregate (**B**) confirming immunonegative epithelioid histiocytes (**A**; arrows) and lymphocytes (**B**). (**C)** IgG4 immunopositive plasma cells (arrows) in the granulation tissue layer functioning as a positive, in-built isotype control, and surrounding stromal immunonegative endothelial, mesenchymal, and lymphoid cells. **(D)** As anticipated, intra-alveolar blood/serum products demonstrate immunopositivity (asterisks) as the normal serum IgG4 level is 8-140 mg/dL. However, the viable intra-alveolar and interstitial cellular components are immunonegative. The clear cytoplasm and hematoxyphylic nuclei (blue) are due to hematoxylin counterstaining. Tissue staining and staining controls obtained from three TB patients (Table S1) were performed at least three times independently post optimization.

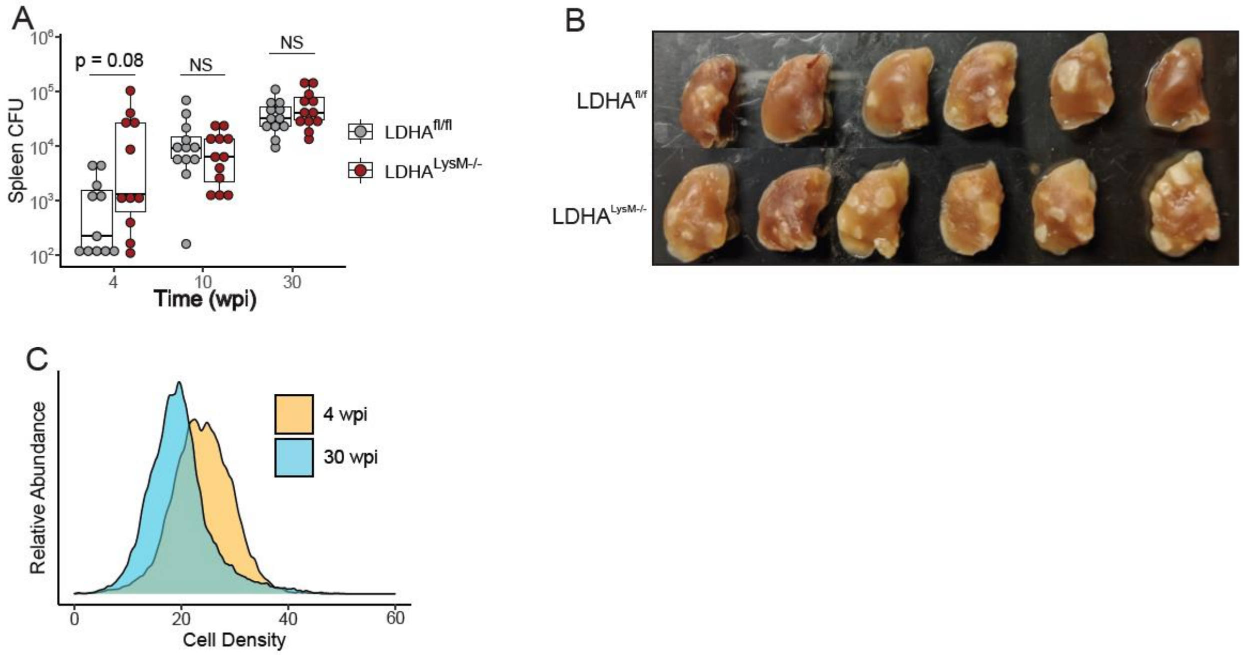

Figure S3. *Ldha*$^{LysM-/-}$ mice are more susceptible to *Mtb* infection. (A) Box plot representing *Mtb* burden in the spleens of mice. Bottom, middle, and top horizontal lines for each condition represent the 25th, 50th, and 75th percentile, respectively, while the whiskers extend from the edge of the box to the most distant point no further than 1.5 times interquartile range. Symbols represent biological replicates pooled from two independent experiments (n ≥ 10/group). **(B)** Gross pathology of formalin-fixed lungs from *Mtb*-infected mice sacrificed at 30 wpi. **(C)** Histograms of cumulative cell density within tissue sections of *Ldha*$^{fl/fl}$ mice at 4 and 30 wpi. Statistical significance in **(A)** was determined by two-sided, two-sample Wilcoxon rank-sum test. Source data are provided as a Source Data file.

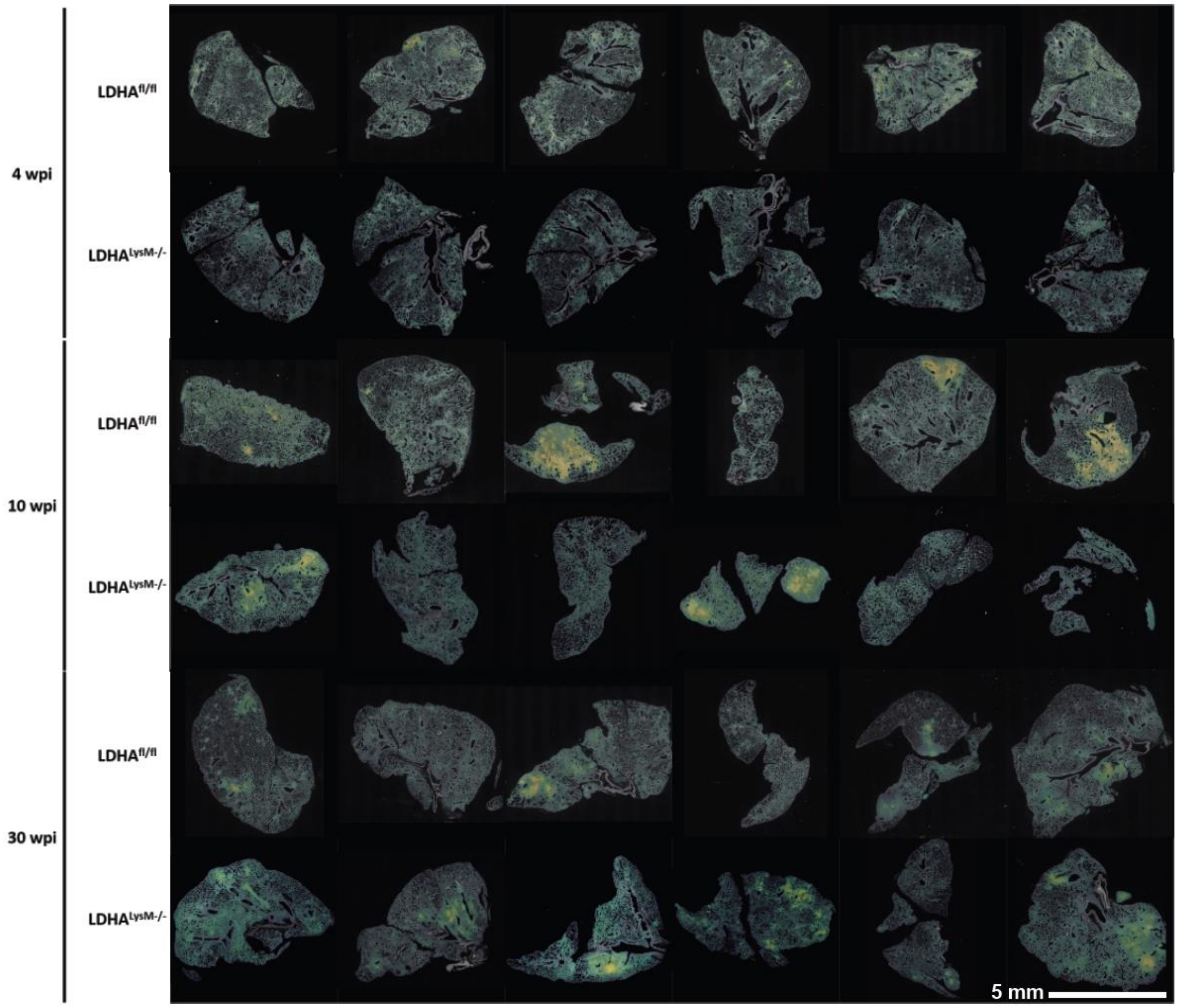

Figure S4. ***Ldha*$^{LysM-/-}$ mice exhibit slow-developing chronic inflammation following infection with *Mtb*.** **(A)** Gray-scale rendering of the optical density of whole-tissue, H&E-stained lung sections from *Mtb*-infected mice. Nuclei were segmented based on optical density and pseudocolored with the viridis color scale based on local cell density, ranging from low desity (dark blue) to medium (green) to high density (yellow).

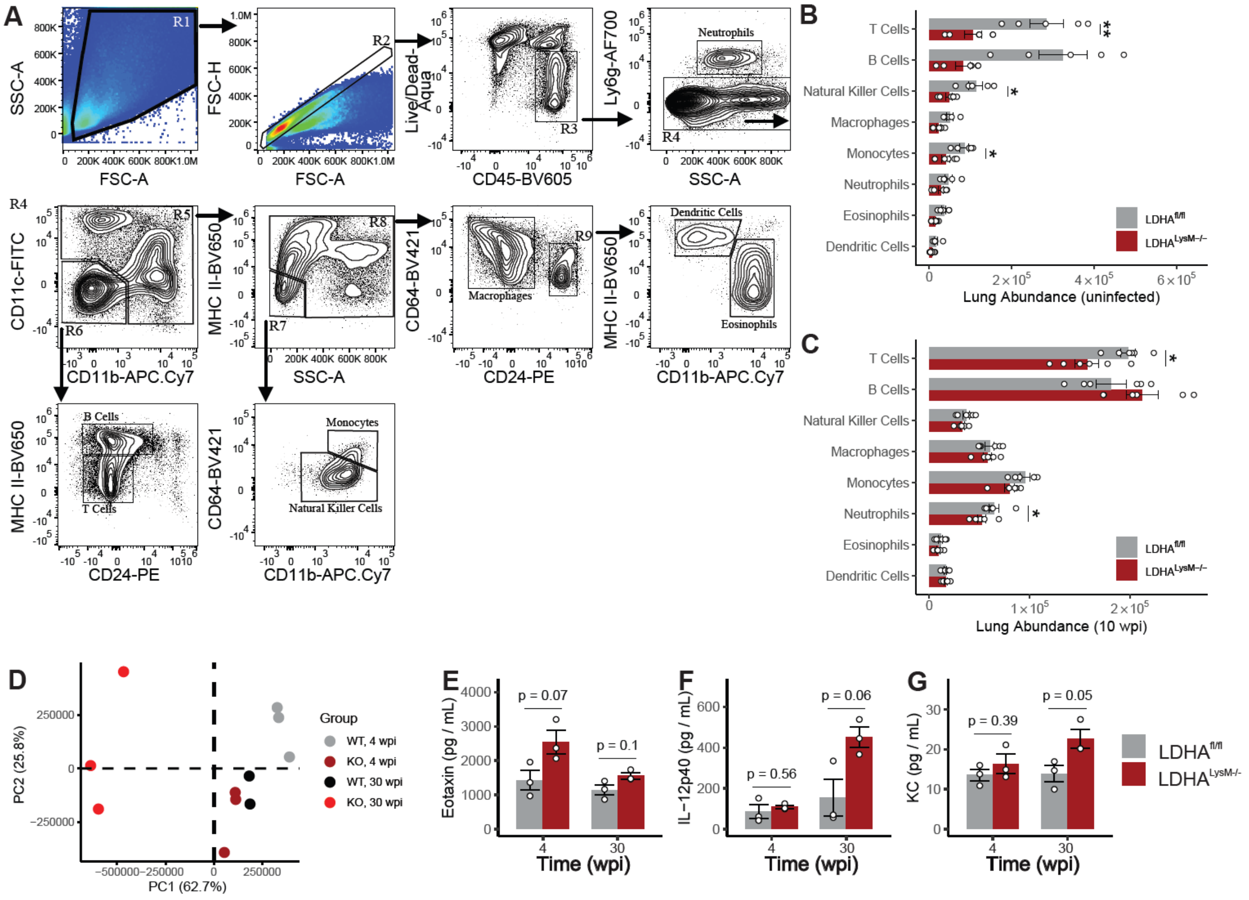

Figure S5. ***Ldha*$^{LysM-/-}$ mice exhibit dysregulated immunity during *Mtb* infection.** **(A)** Gating strategy for multi-parameter flow cytometry. **(B, C)** Bar graph with error bars depicting the mean ± SEM for the absolute count of the indicated immune cell population in the lungs of *Mtb*-infected mice either uninfected **(B)** or at 10 wpi (C; n = 6/group). **(D)** Principal component analysis of the global transcriptome in the lungs of *Mtb*-infected mice. Symbols represent biological replicates. **(E-G)** Column graphs, error bars, and points representing the mean, SEM, and individual protein expression of biological replicates for the indicated cytokines (n = 3/group). Statistical significance was determined by the two-sided, two-sample Wilcoxon rank-sum test **(B, C)** or two-sided, two-sample t-test not assuming equal variance **(E-G)**. * $p < 0.05$, ** $p < 0.01$. Source data are provided as a Source Data file.

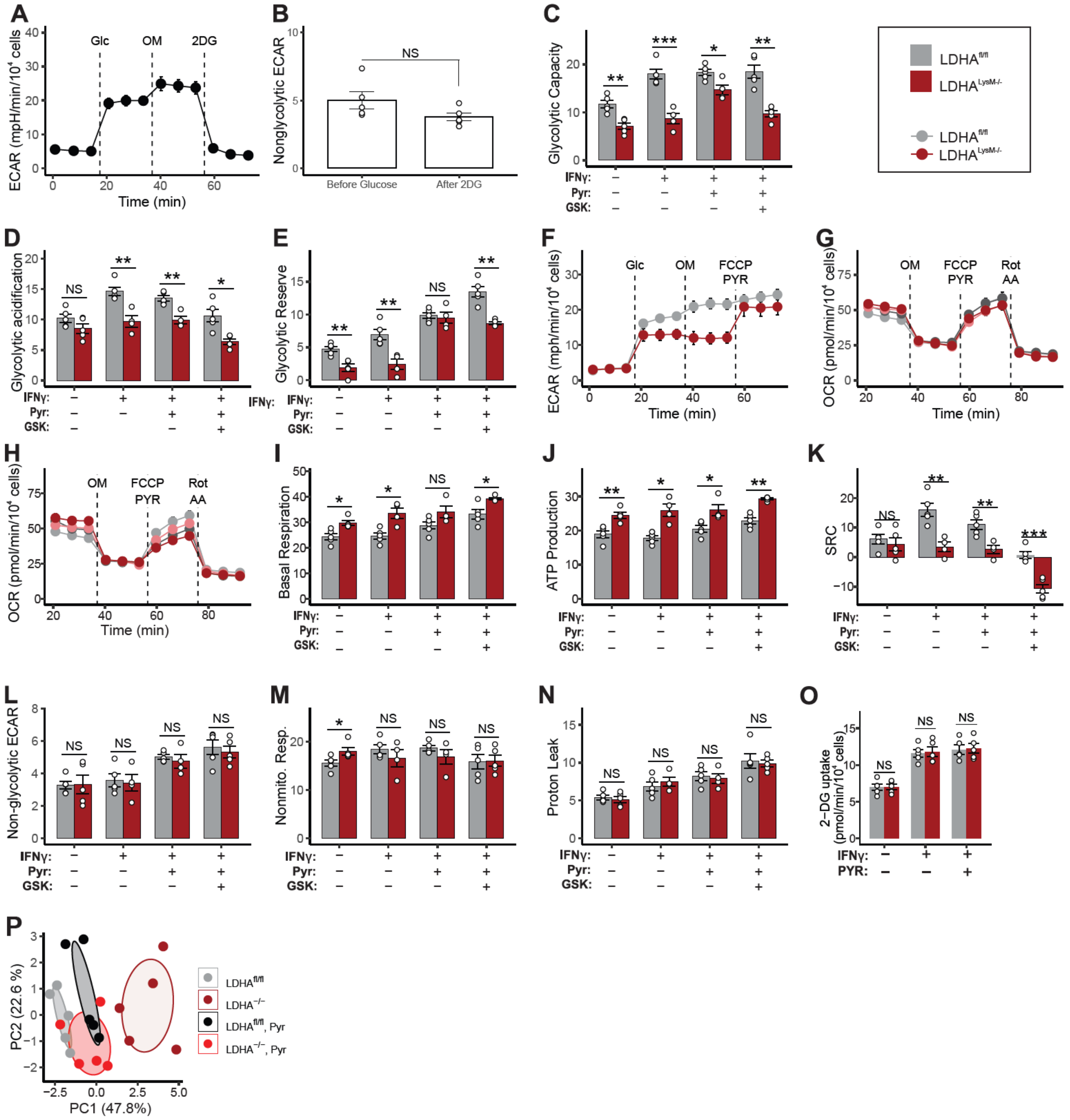

Figure S6. LDHA$^{-/-}$ BMDMs stimulated with IFNγ exhibit diminished glycolysis and increased reliance on OXPHOS. (A) ECAR of BMDMs treated with 10 ng/mL IFNγ. Dashed lines represent the injections of glucose, oligomycin, and 2DG. Symbols and error bars represent mean ±SEM of 5 biological replicates. **(B)** Columns, error bars, and symbols represent the mean, ±SEM, and individual values of biological replicates for non-glycolytic acidification as determined by the 3rd read and 12th read of panel A (n = 5/group). **(C-E)** Columns, error bars, and symbols represent the mean, SEM, and individual values of biological replicates for glycolytic capacity, basal glycolysis, and glycolytic reserve of BMDMs treated as indicated (n = 5/group). **(F)** ECAR of BMDMs treated with 10 ng/mL IFNγ. Dashed lines represent the injections (L to R) of glucose, oligomycin, and FCCP/pyruvate. Symbols and error bars represent mean ± SEM of 5 biological replicates. **(G, H)** OCR of BMDMs treated with **(G)** 10 ng/mL IFNγ and 1 mM pyruvate (solid) or IFNγ alone (light) and **(H)** IFNγ, pyruvate, and GSK 2837808A (solid) or IFNγ alone (light). Dashed lines indicate injections of oligomycin, FCCP/Pyruvate,

and Rotenone/Antimycin A. Points and error bars represent mean ±SEM of 5 biological replicates. **(I-N)** Columns, error bars, and symbols represent the mean, SEM, and individual values of biological replicates for basal respiration, ATP production by respiration, spare respiratory capacity (SCR), non-glycolytic acidification, nonmitochondrial respiration, and proton leak of BMDMs treated as indicated (n = 5/group). **(O)** Columns, error bars, and symbols represent the mean, SEM, and individual values of biological replicates for 2DG uptake in BMDMs under the indicated treatment conditions (n = 4/group). **(P)** Principal component analysis of the abundance of glycolytic and PPP intermediates in BMDMs under the indicated conditions. Symbols represent biological replicates and ellipses represent 95% confidence intervals for each group. Statistical significance was determined by two-sided, two-sample t-test without assuming equal variance **(B-E, I-O)**. * $p < 0.05$, ** $p < 0.01$, *** $p < 0.001$; exact p-values for each comparison are listed in supplemental data. Source data are provided as a Source Data file.

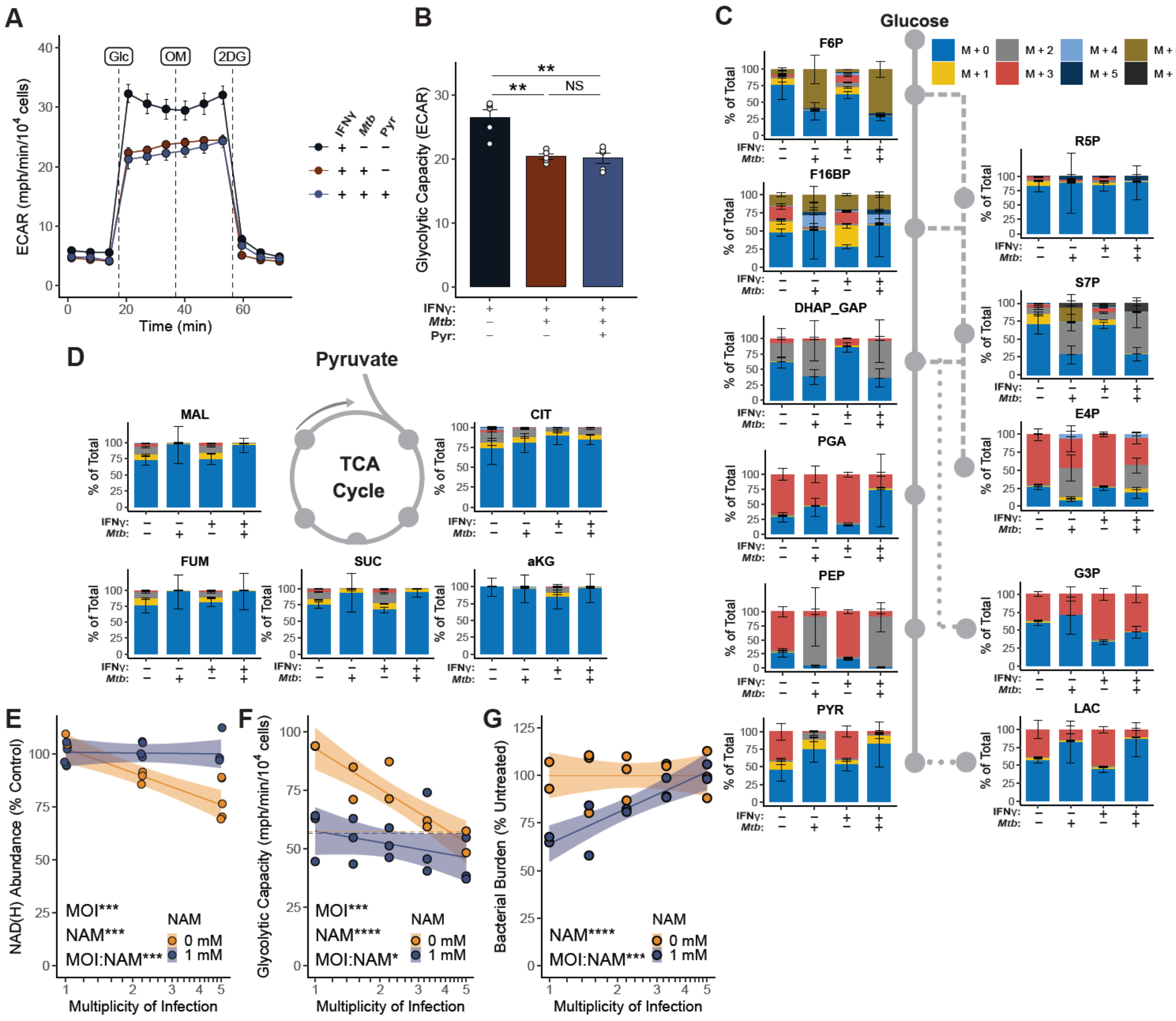

Figure S7. Pyruvate does not rescue *Mtb*-induced glycolytic defect in infected macrophages. (**A**) ECAR profiles for BMDMs exposed to combinations of IFNγ (10 ng/mL), *Mtb* (MOI 5:1), and pyruvate (1 mM) for 18 hours. Dashed lines indicate injections of glucose (Glc), oligomycin (OM), or 2DG. Symbols and error bars represent mean ± SEM of 5 biological replicates. **(B)** Columns, error bars, and symbols representing the mean, SEM, and individual values for the glycolytic capacity determined from the profiles in panel **(A)**(n = 5/group). Statistical significance was determined by two-sided, two-sample t-test without assuming equal variance. **(C, D)** Stacked column graphs corresponding to 100% of the total abundance for each metabolite (shown in Figure 4K), where the proportion of each mass isotopologue is represented by color and error bars represent the SEM for the group of biological replicates (n = 4/group). **(E–G)** Individual values fit with a linear regression (solid line; further described in the methods) and 95% confidence interval (shaded region) for **(E)** NAD(H) abundance, **(F)** glycolytic capacity determined by XF analysis, and **(G)** bacterial burden in human monocyte-derived macrophages (hMDMs) at the indicated multiplicity of infection and nicotinamide (NAM) concentration (yellow = 0 mM, blue = 1 mM). Dotted lines in **(F)** indicate the mean glycolytic capacity of uninfected hMDMs. Statistical significance for each term in the linear regression was determined by ANCoVA (two-sided) and is indicated in the bottom left of each graph. MOI as a standalone factor was excluded from the analysis **(G)**, as bacterial

burden was represented as a percentage of untreated hMDMs within each MOI (n ≥ 10/group). Exact p-values for each comparison are listed in supplemental data. Source data are provided as a Source Data file.
