# Supplementary material for: NAD(H) homeostasis underlies host protection mediated by glycolytic myeloid cells in tuberculosis
Source: Nat Commun. 2023 Sep 6;14:5472. doi: 10.1038/s41467-023-40545-x (PMC10482943; doi:10.1038/s41467-023-40545-x)
Supplement: Supplementary file 3 — Description of Additional Supplementary Files [file 41467_2023_40545_MOESM3_ESM.pdf]

Description of Additional Supplementary Files

File Name: Supplementary Data 1
Description: p-values for comparisons made throughout the manuscript.
