# Supplementary material for: NAD(H) homeostasis underlies host protection mediated by glycolytic myeloid cells in tuberculosis
Source: Nat Commun. 2023 Sep 6;14:5472. doi: 10.1038/s41467-023-40545-x (PMC10482943; doi:10.1038/s41467-023-40545-x)
Supplement: Supplementary file 5 — Reporting Summary [file 41467_2023_40545_MOESM5_ESM.pdf]

nature portfolio

Corresponding author(s): Andries J. C. Steyn

Last updated by author(s): Jul 23, 2023

Reporting Summary

Nature Portfolio wishes to improve the reproducibility of the work that we publish. This form provides structure for consistency and transparency in reporting. For further information on Nature Portfolio policies, see our Editorial Policies and the Editorial Policy Checklist.

Statistics

For all statistical analyses, confirm that the following items are present in the figure legend, table legend, main text, or Methods section.

n/a	Confirmed	
☐	☒	The exact sample size (*n*) for each experimental group/condition, given as a discrete number and unit of measurement
☐	☒	A statement on whether measurements were taken from distinct samples or whether the same sample was measured repeatedly
☐	☒	The statistical test(s) used AND whether they are one- or two-sided<br>*Only common tests should be described solely by name; describe more complex techniques in the Methods section.*
☐	☒	A description of all covariates tested
☐	☒	A description of any assumptions or corrections, such as tests of normality and adjustment for multiple comparisons
☐	☒	A full description of the statistical parameters including central tendency (e.g. means) or other basic estimates (e.g. regression coefficient) AND variation (e.g. standard deviation) or associated estimates of uncertainty (e.g. confidence intervals)
☐	☒	For null hypothesis testing, the test statistic (e.g. F, t, r) with confidence intervals, effect sizes, degrees of freedom and P value noted<br>*Give P values as exact values whenever suitable.*
☒	☐	For Bayesian analysis, information on the choice of priors and Markov chain Monte Carlo settings
☒	☐	For hierarchical and complex designs, identification of the appropriate level for tests and full reporting of outcomes
☒	☐	Estimates of effect sizes (e.g. Cohen's *d*, Pearson's *r*), indicating how they were calculated

Our web collection on statistics for biologists contains articles on many of the points above.

Software and code

Policy information about availability of computer code

Data collection	For data collection:<br>NDP.View2 (Hamamatsu)<br>Attune NxT software version 5.2 (ThermoFisher Scientific)<br>NextSeq software version 4.2.0 (Illumina)<br>xPONENT software version 5.1.0 (Luminex)<br>Wave software version 6.2.1.53 (Agilent)<br>Gen5 software version 3.12.08 (Biotek).
Data analysis	For data analysis:<br>Fiji/ImageJ version 1.52p<br>QuPath version 0.4.3<br>FlowJo version 10 (TreeStar, Inc.)<br>R statistical software version 4.0.2<br>Rstudio version 1.3<br>Skyline software version 4.1.0.<br>HTSeq-count version 0.13.5<br>STAR alignment software version 2.7.3a

For manuscripts utilizing custom algorithms or software that are central to the research but not yet described in published literature, software must be made available to editors and reviewers. We strongly encourage code deposition in a community repository (e.g. GitHub). See the Nature Portfolio guidelines for submitting code & software for further information.

Data

Policy information about availability of data

All manuscripts must include a data availability statement. This statement should provide the following information, where applicable:

- Accession codes, unique identifiers, or web links for publicly available datasets
- A description of any restrictions on data availability
- For clinical datasets or third party data, please ensure that the statement adheres to our policy

RNA sequencing data generated in this study have been deposited in the NCBI GEO Repository under accession code GSE234115 [ncbi.nlm.nih.gov/geo/]. Processed RNA sequencing data and all other source data are included with this manuscript.

Research involving human participants, their data, or biological material

Policy information about studies with human participants or human data. See also policy information about sex, gender (identity/presentation), and sexual orientation and race, ethnicity and racism.

Reporting on sex and gender	No information regarding sex and gender was collected from human participants. Sex and gender are not related to our hypothesis, and only within-subject comparisons were made.
Reporting on race, ethnicity, or other socially relevant groupings	No information regarding race, ethnicity, and other socially relevant groupings was collected from human participants. These factors are not related to our hypothesis, and only within-subject comparisons were made.
Population characteristics	No demographic information was collected from human participants. These factors are not related to our hypothesis, and only within-subject comparisons were made.
Recruitment	For the collection of human surgical tissue remnants for analysis of lung pathology, written informed consent was obtained from patients recruited from King DinuZulu Hospital Complex, a tertiary center for TB patients in Durban, South Africa.<br><br>For the collection of human blood, flyers were posted in academic buildings on campus, and participants called researchers to volunteer. Participants were compensated $20 for one donation.
Ethics oversight	The collection of human surgical tissue remnants for analysis of lung pathology was approved by the University of KwaZulu-Natal Biomedical Research Ethics Committee (BREC, Class approval study number BCA 535/16). Patients undergoing lung resection for TB, their study protocol, associated informed consent documents, and data collection tools were approved by the UKZN BREC (Study ID: BE 019/13). Written informed consent was obtained from patients recruited from King DinuZulu Hospital Complex, a tertiary center for TB patients in Durban, South Africa.<br><br>Collection of human blood was approved by the Institutional Review Board at the University of Alabama at Birmingham (IRB-300004485). Written informed consent was received from all participants prior to inclusion in the study.

Note that full information on the approval of the study protocol must also be provided in the manuscript.

Field-specific reporting

Please select the one below that is the best fit for your research. If you are not sure, read the appropriate sections before making your selection.

☒ Life sciences ☐ Behavioural & social sciences ☐ Ecological, evolutionary & environmental sciences

For a reference copy of the document with all sections, see nature.com/documents/nr-reporting-summary-flat.pdf

Life sciences study design

All studies must disclose on these points even when the disclosure is negative.

Sample size	For in vivo experiments, sample size was determined by power analysis to limit the number of animals used in a given experiment. Otherwise, sample size was guided by standards in the field (default n = 3-6 / experimental condition) and technical considerations.
Data exclusions	For high-paremeter data (e.g. RNA sequencing, metabolomics), samples were excluded when the majority of variance within an experiment was attributable to an individual data point (i.e outlier) as determined by principle component analysis. For other experiments, samples were only excluded in the event of a known technical deviation or an identifiable, unrelated effect (e.g. batch effect, position/edge effect).
Replication	Experimental findings were replicated using biological replicates within and between independent experiments. In almost all cases, findings were validated in at least two independent experiments, with the exception being experiments that were deemed too time- or cost-intensive (e.g., metabolomics, survival) and could be supported and extended by complimentary techniques (e.g., extracellular flux analysis, organ burden).
Randomization	Within-sample comparisons were made in almost all cases; otherwise, mice were randomized to the different experimental conditions.

Blinding	Experimenters were blinded to treatment condition for the counting of colonies; however, blinding was not always technically feasible. In these instances, samples were processed and/or positioned in an intercalated fashion to limit bias from group/batch effects. Subsequent data acquisition and analysis was performed in an automated fashion to reduce experimenter bias.

Reporting for specific materials, systems and methods

We require information from authors about some types of materials, experimental systems and methods used in many studies. Here, indicate whether each material, system or method listed is relevant to your study. If you are not sure if a list item applies to your research, read the appropriate section before selecting a response.

Materials & experimental systems

n/a	Involved in the study
☐	☒ Antibodies
☒	☐ Eukaryotic cell lines
☒	☐ Palaeontology and archaeology
☐	☒ Animals and other organisms
☒	☐ Clinical data
☒	☐ Dual use research of concern
☒	☐ Plants

Methods

n/a	Involved in the study
☒	☐ ChIP-seq
☐	☒ Flow cytometry
☒	☐ MRI-based neuroimaging

Antibodies

Antibodies used	BioLegend antibodies (all anti-mouse):<br>PerCP-Cy5.5 anti-Ly6C (cat # 128012), Clone HK1.4, Lot B250462<br>APC-Cy7 anti-CD11b (cat # 101226), Clone M1/70, Lot B273857<br>AF700 anti-Ly6G (cat # 127622), Clone 1A8, Lot B266371<br>PE-Cy7 anti-F4/80 (cat # 123114), Clone BM8, Lot B265636<br>BV785 anti-CD11c (cat # 117336), Clone N418, Lot B265353<br>BV605 anti-CD45 (cat # 103155), Clone 30-F11, Lot 263874<br>BV421 anti-CD64 (cat # 139309), Clone X54-5/7.1, Lot B254355<br>BV650 anti-I-A/I-E (cat # 107641), Clone M5/114.15.2, Lot B266132<br>PE anti-CD24 (cat # 138503), Clone 30-F1, Lot B264401<br><br>ProteinTech antibodies (anti-mouse):<br>anti-LDHA (cat # 19987-1-AP), rabbit polyclonal IgG<br><br>Abcam antibodies (anti-human):<br>anti-lactate dehydrogenase (cat # ab125683), rabbit polyclonal IgG<br><br>LSBio antibodies (anti-human):<br>anti-IgG4 (LS-C70325), mouse monoclonal IgG1
Validation	Antibodies were validated by their respective manufacturers to ensure specific binding activity and lot-to-lot consistency. Additional details may be found on the manufacturers' website by searching for the respective catalog number listed above.

Animals and other research organisms

Policy information about studies involving animals; ARRIVE guidelines recommended for reporting animal research, and Sex and Gender in Research

Laboratory animals	LysM+/+:Ldhafl/fl (Ldhafl/fl) and LysM+/cre:Ldhafl/fl (LdhaLysM-/-) mice on a C57BL/6 background were originally sourced from an independent investigator and maintained in pathogen-free facilities. C57BL/6J mice were obtained from Jackson Laboratory. All studies used sex- and age-matched ittermates of both sexes. Same-sex littermates were cohoused until the start of experiments. For in vivo experiments, mice were aged 8-12 weeks old at the start. For ex vivo generation of BMDMs, mice aged 8-16 weeks old were used. At the start of in vivo experiments, mice were housed under ABSL-3 conditions and monitored daily. Housing conditions:light/dark cycle: 12h/12h with lights on from 7AM - 7PM. Ambient temperature was approximately 72 F with relative humidity of approximately 50%. All procedures and protocols were approved by the Institutional Animal Care and Use Committee of the University of Alabama at Birmingham.
Wild animals	No wild animals were used.
Reporting on sex	A balanced combination of male and female animals was used in all experiments--in vivo and ex vivo. Sex of an animal was only indicated if sex influenced the dependent variable (e.g. lung weight); otherwise, animals were analyzed in aggregate and no subgroup analyses were reported.
Field-collected samples	No field-collected samples were used.

Ethics oversight | All procedures were conducted according to NIH guidelines and and were approved by the Institutional Animal Care and Use Committee of the University of Alabama at Birmingham.

Note that full information on the approval of the study protocol must also be provided in the manuscript.

Flow Cytometry

Plots

Confirm that:

☒ The axis labels state the marker and fluorochrome used (e.g. CD4-FITC).

☒ The axis scales are clearly visible. Include numbers along axes only for bottom left plot of group (a 'group' is an analysis of identical markers).

☒ All plots are contour plots with outliers or pseudocolor plots.

☒ A numerical value for number of cells or percentage (with statistics) is provided.

Methodology

Sample preparation	Mice were euthanized at indicated timepoints according to IACUC-approved protocols. Immediately following sacrifice and thoracotomy, the pulmonary vasculature was perfused with PBS via the right ventricle. Lobes taken for cytometric analysis were minced in Dulbecco's Modified Eagle Medium and incubated with Liberase (Roche) 2 mg/mL at 37 °C for 30 minutes. The resulting cell suspension was passed 5 times through a 20-gauge needle, 5 times through a 23-gauge needle and filtered through a cell-strainer with a 40 µm pore size. Cells were washed once in PEB buffer and stained with fixable live/dead stain for 30 minutes on ice. Cells were washed in PEB and stained for the indicated surface markers by staining cells with fluorophore-conjugated antibodies for 30 minutes at 4 °C. Cells were then fixed in 4% paraformaldehyde for 30 minutes, resuspended in PBS, and stored at 4 °C in the dark and analyzed the next day.
Instrument	Flow cytometry acquisition was performed using an Attune NxT cytometer (Thermo Fisher Scientific).
Software	Acquisition was accomplished with Attune NxT software version 5.2 (ThermoFisher Scientific), and analysis was performed with FlowJo software v10 (Tree Star, Inc.).
Cell population abundance	Approximately 100,000 - 300,000 events of interest (single, live, CD45-positive cells) were captured per sample.
Gating strategy	We employed a gating strategy for the flow-cytometric phenotyping of immune cells in the inflamed and non-inflamed murine lung (Yu et al., PLOS One, 2016; PMID: 26938654), and we have provided our application of the gating strategy in Figure S5A of the manuscript.

☒ Tick this box to confirm that a figure exemplifying the gating strategy is provided in the Supplementary Information.
